# Influenza Forecasting in Human Populations: A Scoping Review

**DOI:** 10.1371/journal.pone.0094130

**Published:** 2014-04-08

**Authors:** Jean-Paul Chretien, Dylan George, Jeffrey Shaman, Rohit A. Chitale, F. Ellis McKenzie

**Affiliations:** 1 Division of Integrated Biosurveillance, Armed Forces Health Surveillance Center, Silver Spring, Maryland, United States of America; 2 Division of Analytic Decision Support, Biomedical Advanced Research and Development Authority, Department of Health and Human Services, Washington, DC, United States of America; 3 Department of Environmental Health Sciences, Mailman School of Public Health, Columbia University, New York, New York, United States of America; 4 Fogarty International Center, National Institutes of Health, Bethesda, Maryland, United States of America; University of Massachusetts, United States of America

## Abstract

Forecasts of influenza activity in human populations could help guide key preparedness tasks. We conducted a scoping review to characterize these methodological approaches and identify research gaps. Adapting the PRISMA methodology for systematic reviews, we searched PubMed, CINAHL, Project Euclid, and Cochrane Database of Systematic Reviews for publications in English since January 1, 2000 using the terms “influenza AND (forecast* OR predict*)”, excluding studies that did not validate forecasts against independent data or incorporate influenza-related surveillance data from the season or pandemic for which the forecasts were applied. We included 35 publications describing population-based (N = 27), medical facility-based (N = 4), and regional or global pandemic spread (N = 4) forecasts. They included areas of North America (N = 15), Europe (N = 14), and/or Asia-Pacific region (N = 4), or had global scope (N = 3). Forecasting models were statistical (N = 18) or epidemiological (N = 17). Five studies used data assimilation methods to update forecasts with new surveillance data. Models used virological (N = 14), syndromic (N = 13), meteorological (N = 6), internet search query (N = 4), and/or other surveillance data as inputs. Forecasting outcomes and validation metrics varied widely. Two studies compared distinct modeling approaches using common data, 2 assessed model calibration, and 1 systematically incorporated expert input. Of the 17 studies using epidemiological models, 8 included sensitivity analysis. This review suggests need for use of good practices in influenza forecasting (e.g., sensitivity analysis); direct comparisons of diverse approaches; assessment of model calibration; integration of subjective expert input; operational research in pilot, real-world applications; and improved mutual understanding among modelers and public health officials.

## Introduction

Seasonal influenza epidemics caused by influenza A and B viruses occur annually during the winter in temperate regions, resulting in around 3–5 million cases of severe illness and 250,000–500,000 deaths worldwide each year [1]. In contrast to seasonal influenza, novel influenza A strains capable of sustained person-to-person transmission arise occasionally. These novel strains may evade existing antibody immunity and give rise to pandemic outbreaks. For example, the 1918 pandemic caused around 20–40 million deaths [2], while pandemics in 1957 and 1968 involved many infections but fewer deaths than in the 1918 pandemic. A 2009 pandemic strain, influenza A(H1N1)pdm09, continues to circulate as a seasonal virus.

Accurate forecasts of influenza activity based on predictive models could facilitate key preparedness actions, such as public health surveillance, development and use of medical countermeasures (e.g., vaccine and antiviral drugs), communication strategies, deployment of Strategic National Stockpile assets in anticipation of surge demands (e.g., ventilators), and hospital resource management (e.g., for staf and beds). Early in a potential pandemic, forecasts of international spread could help guide public health actions globally.

Previous reviews have assessed influenza modeling (e.g., [3]–[6]), but to our knowledge only one focused specifically on the use of models to forecast influenza activity, as opposed to other important applications of influenza modeling (such as improving understanding of the epidemiological dynamics or evaluating control strategies). This recent review, by Nsoesie et al. [7], identified 16 studies that aimed to forecast influenza outbreaks at local, regional, national, or global level. To more systematically characterize influenza forecasting methods and applications, and identify promising approaches and research gaps, we conducted a scoping review of the peer-reviewed influenza forecasting literature. We assess differences in methodological approaches and provide recommendations for future influenza forecasting models.

## Materials and Methods

We adapted the PRISMA methodology [8] for our scoping review. In contrast to a systematic review, which focuses on a well-defined research question and may include a narrow range of study designs, a scoping review addresses broader topics and may include various study designs [9]. We included studies that described methods to forecast future influenza activity in human populations using dynamic influenza-related surveillance data, and that tested the forecasting approach against independent data (real or simulated). We defined “dynamic” data as data collected during an epidemic or pandemic to make predictions about its subsequent course. We excluded studies that predicted current influenza activity not observed at the time of prediction because of reporting delays (sometimes called “nowcasting”).

We searched PubMed, CINAHL, Project Euclid, and Cochrane Database of Systematic Reviews for publications in English since January 1, 2000 using the terms “influenza AND (forecast* OR predict*)” in any field, and analyzed abstracts of returned publications to identify candidates for full-text review (i.e., studies for which inclusion criteria were met or for which it was not possible to determine whether inclusion criteria were met). We also manually searched the reference lists of included papers, our bibliographies, and the *International Journal of Forecasting*, and considered recommendations of colleagues.

We abstracted the data as follows. One of us conducted the literature search on December 4, 2013 and determined which publications to include based on the abstract or full text. For included publications, the reviewer recorded the geographic setting, data timeframe, whether the focus was seasonal or pandemic influenza, details of the input data and analytical methods, and reported forecast accuracy. Another reviewer independently abstracted the data from selected publications. The two reviewers resolved discrepancies through consensus.

## Results

**Figure 1:**
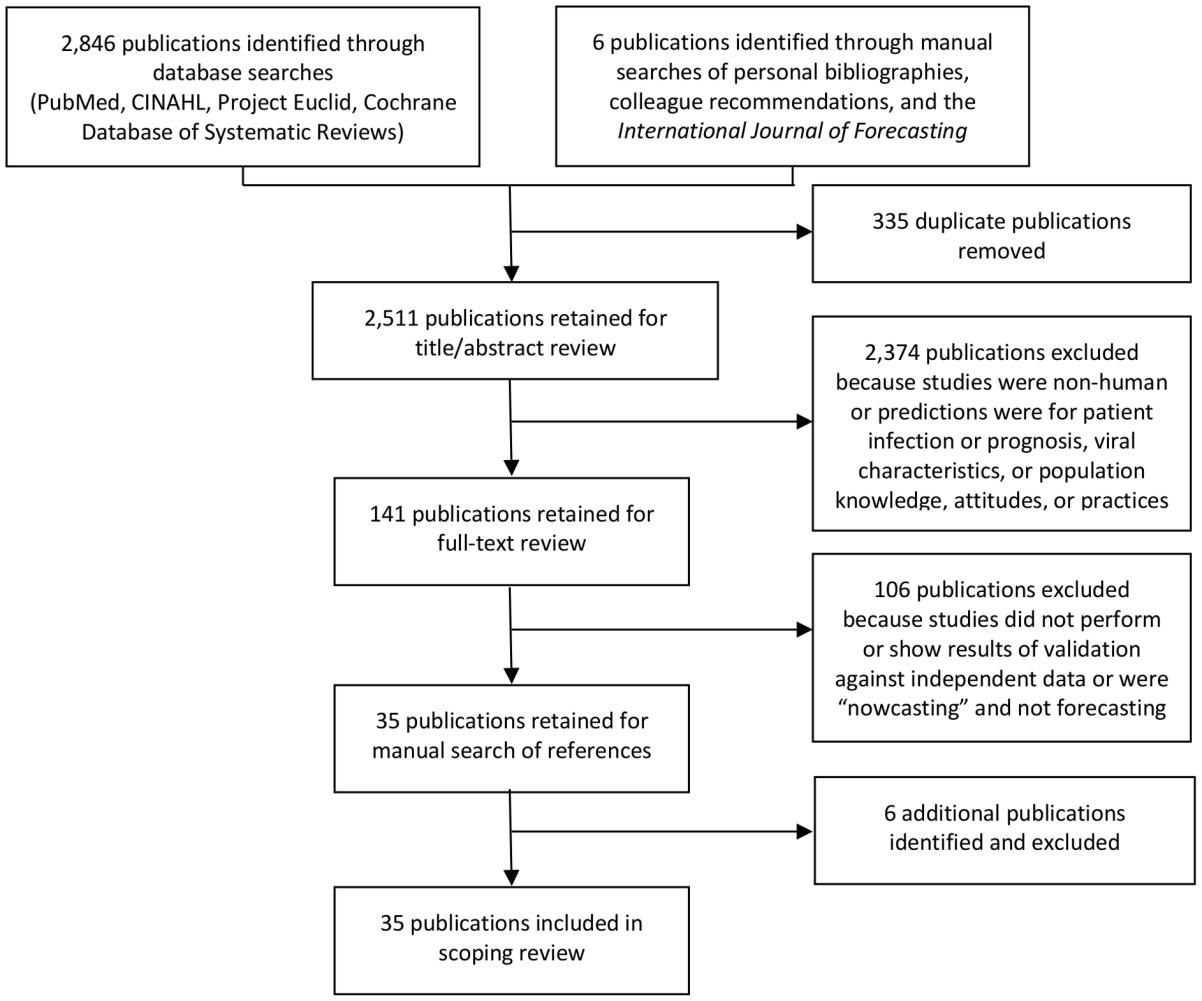
Literature search flow.

We included 35 publications in the review [10]–[44] (Figure 1). Twenty six (74%) of the studies were published in 2009 or later, with more than one-third published in 2012 or 2013. The studies fell into 3 categories based on the epidemiological application: population-based seasonal influenza forecasting (N = 27 publications), medical facility-based forecasting of patient counts for seasonal or pandemic influenza (N =  4), and regional or global spread forecasting for pandemic influenza (N = 4) (Table 1). Most studies included areas of North America (N = 15) or Europe (N = 14 publications), while a few included areas in the Asia-Pacific region (N = 4) or had global scope (N = 3) (Table 1).

**Table 1:** Overview of influenza forecasting studies.

Ref.	Influenza Application	Setting	Forecast Type	Forecasting Method
***Population-based forecasting studies***				
[10]	Seasonal	United States	Temporal	Mechanistic (compartmental model)
[11]	Seasonal	Seattle	Temporal	Mechanistic (ABM)
[12]	Seasonal	Montreal	Temporal	Mechanistic (ABM)
[13]	Unspecified	Montgomery Co., VA; Seattle; Miami	Temporal	Mechanistic (ABM)
[14]	Pandemic (2009)	New Zealand	Temporal	Mechanistic (compartmental model)
[15]	Seasonal	Germany	Spatial-temporal	Statistical (time series model)
[16]	Seasonal	New York City	Temporal	Mechanistic (compartmental model)
[17]	Seasonal	Slovenia	Temporal	Statistical (GLM, regression tree)
[18]	Pandemic (2009)	Italy	Temporal	Mechanistic (ABM)
[19]	Pandemic (2009)	London	Temporal	Mechanistic (compartmental model)
[20]	Seasonal	United States	Temporal	Statistical (GLM)
[21]	Pandemic (2009)	Japan	Temporal	Mechanistic (compartmental model)
[22]	Unspecified	Los Angeles; New York; Seattle	Temporal	Statistical (classification)
[23]	Pandemic (2009)	Japan	Temporal	Mechanistic (compartmental model)
[24]	Seasonal	Germany	Spatial-temporal	Statistical (time series model)
[25]	Pandemic (2009)	Singapore	Temporal	Mechanistic (compartmental model)
[26]	Seasonal	Hong Kong; Maricopa Co., AZ	Temporal	Statistical (time series model)
[27]	Seasonal	2 US jurisdictions (not identified)	Temporal	Statistical (Bayesian network)
[28]	Seasonal	United Kingdom (boarding school)	Temporal	Mechanistic (compartmental model)
[29]	Seasonal	Sweden	Temporal	Statistical (GLM)
[30]	Seasonal	United Kingdom	Temporal	Statistical (time series model)
[31]	Pandemic (1918, 1957, 1968)	United Kingdom	Temporal	Mechanistic (compartmental model)
[32]	Seasonal	Iowa	Temporal	Statistical (prediction market)
[33]	Seasonal	Massachusetts	Temporal	Statistical (Bayesian network)
[34]	Seasonal	United States, United Kingdom	Temporal	Mechanistic (compartmental model)
[35]	Seasonal	France	Spatial-temporal	Statistical (time series model)
[36]	Seasonal	Scotland	Temporal	Statistical (GLM)
***Facility-based forecasting studies***				
[37]	Seasonal	Baltimore	Temporal	Statistical (time series model)
[38]	Pandemic (2009)	Washington, DC	Temporal	Statistical (time series model)
[39]	Seasonal	Baltimore	Temporal	Statistical (time series model)
[40]	Seasonal	Barcelona	Temporal	Statistical (time series model)
***Regional or global pandemic spread forecasting studies***				
[41]	Pandemic (2009)	Global	Spatial-temporal	Mechanistic (compartmental model)
[42]	Pandemic (2009)	Global	Spatial-temporal	Mechanistic (compartmental model)
[43]	Pandemic (2009)	Europe	Spatial-temporal	Mechanistic (ABM)
[44]	Pandemic (2009)	Global	Spatial-temporal	Statistical (survival analysis)

GLM, generalized linear model; ABM, agent-based model.

Twenty-eight studies employed temporal forecasting without a spatial component, while 7 made forecasts in time and space (Table 1). The studies used diverse forecasting methods, with 18 using statistical approaches without models for epidemiological processes and 17 employing epidemiological models (Table 1).

Among the statistical approaches, methods included time series models (N = 9 publications), generalized linear models (N = 4), Bayesian networks (N = 2), classification methods (N = 2), survival analysis (N = 1), and a prediction market (N = 1) (Table 1). The mechanistic approaches included compartmental models, which model transitions across various sub-populations (susceptible-infectious-removed [SIR] models and variants) (N = 12 publications); and agent-based models (ABMs), which model exposure, infection, transmission and behaviors for each individual in the population (N = 5) (Table 1).

Several studies coupled mechanistic models with methods to update parameter estimates and forecasts as new influenza-related surveillance data becomes available. Nsoesie et al. [11], [13] developed a simulation-optimization algorithm for their ABM, which iteratively proposes estimates of key epidemiological parameters, uses those estimates to simulate the future course of the epidemic, and compares observed surveillance data to forecasts to revise the parameter estimates. Ong et al. [25] and Shaman et al. [10], [16] used data assimilation techniques to incorporate influenza-related surveillance data into their compartmental models and update parameter estimates and forecasts.

The studies used dynamic virological (N = 14), syndromic influenza-like illness (ILI; N = 13) and other influenza-related surveillance data to forecast influenza activity (Table 2). Birrell et al. [19] included serological data to model pre-existing immunity, as well as virological and syndromic data. Four studies included internet search query data (Google Flu Trends) [10], [11], [16], [37]. Six studies considered meteorological data [10], [16], [26], [37]–[39], with 3 including the meteorological predictors in the final forecasting model [10], [16], [26].

**Table 2:** Dynamic surveillance data used in forecasting studies.

Ref.	Data Timeframe	Influenza Data			Meteorological Data
		Virology	ILI	Other	
***Population-based forecasting studies***					
[10]	2012-3	*	*	Google Flu Trends	*
[11]	2007-8, 2012-3			Google Flu Trends	
[12]	2001-6	*			
[13]	NA (simulated data)			Simulated incidence	
[14]	2009	*			
[15]	2001-8	*			
[16]	2003-5, 2007-9			Google Flu Trends	*
[17]	2006-2009		*	Medication sales	
[18]	2009		*		
[19]	2009-10	*	*	Serology	
[20]	1997-2009	*	*		
[21]	2009-10		*		
[22]	NA (simulated data)			Simulated incidence	
[23]	2009-10			Medication prescriptions	
[24]	2001-8	*			
[25]	2009-10		*		
[26]	2004-9	*			*
[27]	2003		*		
[28]	1978			Confined to bed	
[29]	1998-2006	*			
[30]	1992-2005	*			
[31]	1918-9, 57-8, 68–70		*	Influenza deaths	
[32]	2004-5			Prediction market trades	
[33]	1998-2000		*		
[34]	2001-2 (US), 2003-4 (UK)	*			
[35]	1984-2002		*		
[36]	1972-99		*		
***Facility-based forecasting studies***					
[37]	2004-11	*		Google Flu Trends	*
[38]	2009-11	*			*
[39]	2002–2008	*			*
[40]	2004–2008		*		
***Regional or global pandemic spread forecasting studies***					
[41]	2009			Pandemic emergence	
[42]	2009–10			Pandemic emergence	
[43]	2009			Pandemic emergence	
[44]	2009			Pandemic emergence	

ILI, influenza-like illness.

While most studies reported various modeling outcomes, such as ILI time series, the specific outcomes used in model validation varied. Among the 27 population-based forecasting studies, 16 used weekly predictions of weekly incidence 1 or more weeks into the future in the validation (Table 3). Nine studies predicted the timing of the epidemic peak or incidence at the peak; all performed validation using at least some forecasts made at least 4 weeks before the actual peak [10]–[13], [16]–[18], [29], [31]. The facility-based forecasting studies used 1-step-ahead [37]–[39] or *n*-step-ahead [40] predictions of visit counts over step sizes of 1 day [40] to 1 month [39]. The regional or global pandemic spread forecasting studies used early data from the 2009 influenza A(H1N1)pdm09 pandemic to predict outcomes at national level across countries, including pandemic arrival, and peak incidence and time of peak.

**Table 3:** Forecast outcomes used in model validation.

Outcome	Number of studies (refs.)
***Population-based forecasting studies***	
Weekly incidence	16 [12], [13], [15], [17]–[19], [21], [23]–[26], [30], [32]–[35]
Daily incidence	3 [14], [27], [28]
Peak time and/or incidence	9 [10]–[13], [16]–[18], [29], [31]
Cumulative incidence	3 [13], [20], [36]
Epidemic duration	2 [12], [31]
***Facility-based forecasting studies***	
Monthly visits	1 [39]
Weekly visits	1 [40]
Visits over 3 days	1 [38]
Peak visits	1 [37]
***Regional or global pandemic spread forecasting studies***	
Peak incidence (national)	1 [43]
Time of pandemic arrival (national)	1 [44]
Time of peak (national)	2 [42], [43]
Cumulative incidence (U.S.)	1 [41]

The studies used various metrics for validation of forecasts against independent data, with mean (or median) absolute error and mean absolute percent error the most common metrics for forecasts of incidence (i.e., daily, weekly, or monthly incidence; peak incidence; or cumulative incidence) (Table 4; studies forecasting peak week or epidemic duration reported the time difference between predicted and observed values in the validation). Among all studies, only 2 reported accuracy as a function of estimated forecast variance [10], [16].

**Table 4:** Validation metrics used in incidence forecasts.

Metric	Number of studies (refs.)
MAE or MdAE	6 [21], [25], [29], [30], [39], [41]
MAPE	5 [12], [27], [31], [32], [36]
RMSE	5 [13], [26], [35], [38], [39]
Correlation or t-test	5 [13], [20], [27], [33], [35]
95% CI	4 [11], [13], [38], [43]
Scoring rules	2 [15], [24]
Forecast confidence^a^	1[37]
No quantitative metric	8 [14], [17]–[19], [23], [28], [34], [40]

MAE, Mean absolute error; MdAE, Median absolute error; MAPE, Mean absolute percent error; RMSE, Root mean square error.

a Forecast confidence was defined as “the percentage of forecast values within a predefined difference of the actual data during an influenza peak (here chosen as 20% of the mean of the maximal point of the influenza peak).”

Comparing the accuracy of the forecasting applications is difficult because forecasting methods, forecast outcomes, and reported validation metrics varied widely. While many studies compared models with different sets of predictors, only 2 compared distinct modeling approaches. Shaman et al. [10] compared their susceptible-infectious-recovered-susceptible (SIRS) compartmental model, coupled to an ensemble-adjusted Kalman filter (SIRS-EAKF), to various resampling approaches using previous influenza seasons. The SIRS-EAKF model was considerably more accurate in predicting ILI peak week for the 2012–2013 season across 108 US cities. Merler et al. [43] compared the performance of an ABM and a simpler compartmental model in predicting the course of the 2009 influenza A(H1N1)pdm09 pandemic in Europe, and found the simpler model failed to predict pandemic dynamics and attack rate accurately across countries.

Among the 17 studies that used epidemiological models, 8 provided results of sensitivity analysis for clinical, epidemiological, demographic, and other parameters [10], [12], [16], [19], [34], [41]–[43]; however, Nsoese et al. published a sensitivity analysis separately [45] for the ABM used in publications included in this review [11], [13], [22].

## Discussion

This review shows accelerating publication of influenza forecasting methods in recent years. We identified diverse modeling applications to forecast influenza and ILI activity in human populations, including various purely statistical approaches and methods based on mechanistic (i.e., epidemiological) modeling. Most models predicted influenza activity in a specific population, while several others predicted presentations at medical facilities or regional or global pandemic spread. Several models incorporated additional data besides clinical or laboratory-based surveillance data to generate forecasts, including internet search queries and meteorological data. The outcomes predicted and metrics used in validation varied. Most studies using mechanistic models did not present a sensitivity analysis for key epidemiological assumptions.

The review provides an overview and assessment of influenza forecasting, describing current approaches and highlighting research needs for this promising new domain of public health preparedness. Since the focus was on the use of models to forecast influenza activity, we included only studies that validated models against independent data, a crucial part of predictive model development since using the same data for model fitting and testing inflates estimates of predictive skill [46]. This approach complements the review of Nsoesie et al. [7], which did not apply this restriction and provided a more detailed consideration of the outcomes predicted and advantages and disadvantages of the modeling methods employed.

The study has some limitations. We cannot exclude the possibility we failed to identify relevant studies, though we used broad search terms and searched multiple databases, and we would not have identified newer studies described only in conference proceedings, or unpublished studies. Papers correctly (based on our criteria) excluded may yet prove useful for influenza forecasting, and further review of these may suggest new methodologies for generating influenza predictions. The review also cannot serve as a definitive guide to forecasting approaches with greater predictive skill, since settings and methodologies varied widely and only 2 studies [10], [43] compared distinct modeling approaches. We approached the review as a scoping, rather than a systematic, review because of this diversity. Also, the purpose was not to offer detailed critiques of modeling methodologies. Such an assessment would be useful, but we believe that a more broadly-based review of forecasting applications provides necessary context for this and other more focused assessments.

**Figure 2:**
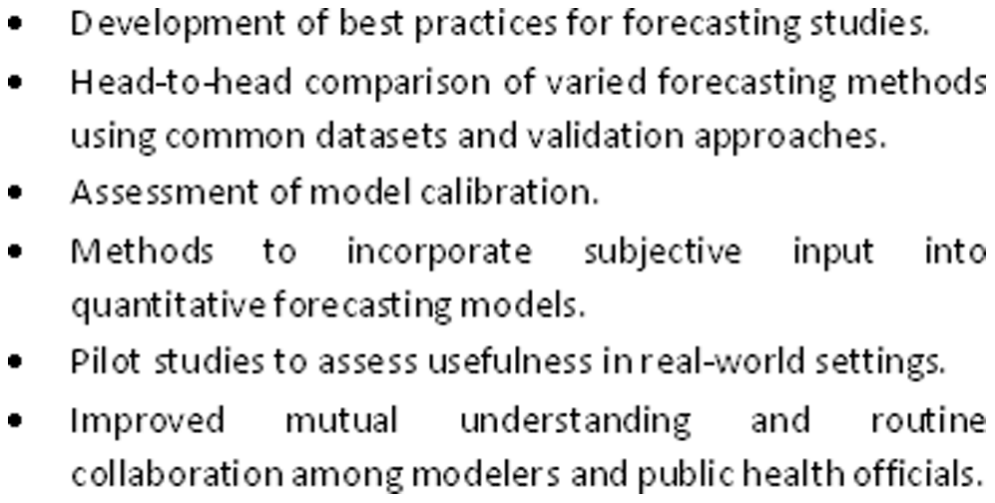
Some needs for advancement of influenza forecasting.

The results suggest several areas of practice and research to advance influenza forecasting in human populations (Figure 2). First, developers of influenza forecasting models and technologies should adhere to good practices in development, implementation, application, and description of epidemiological models. One possible guide [47], developed for veterinary epidemiology but applicable to studies in human populations, provides several recommendations that could facilitate comparison and implementation of influenza forecasting models and technologies. These include use of sensitivity analysis to assess dependence of the model to all chosen parameter values and assumptions, and provision of the computer code implementing the model (in the publication or on request).

However, in light of the methods identified in our review, some modification to these and related guidelines may be appropriate for influenza forecasting. For example, some approaches (e.g., [10], [11], [13], [16], [25]) optimize parameter values iteratively, as part of the forecasting algorithm; model developers do not explicitly assign parameter values or distributions. We encourage developers and users of epidemiological forecasting models to develop common, recommended practices for the field.

Second, there is a need for comparisons of diverse forecasting models using common input data and validation approaches and metrics. While some of the general advantages and disadvantages of various ILI forecasting approaches have been identified [7], direct comparisons would yield insight into methods that perform better than others under particular circumstances. Such initiatives are underway at the Centers for Disease Control and Prevention [48], Intelligence Advanced Research Projects Activity, and Department of Defense, and could help guide future efforts. Head-to-head comparisons of automated detection algorithms to identify disease outbreaks in syndromic surveillance data [49] also could be a useful example for comparing forecasting methods.

Third, assessments of forecasting methods should demonstrate how the accuracy of the method varies and should quantify this variability for use in real-time prediction. That is, it is not sufficient merely to predict an event; the likelihood of that prediction should also be ascribed. This quantification of likelihood, or expected accuracy, mirrors practices used in numerical weather prediction–e.g., a forecast of an 80% chance of rain tomorrow is a highly calibrated prediction of the likelihood of an event. We believe this aspect of model performance – calibration – will be a key consideration for practitioners who might use a forecasting model in an operational setting. Reporting the range or confidence intervals associated with predicted outcomes is essential in validation studies, but this alone does not help a user determine how much certainty a specific forecast warrants.

Fourth, future operational forecasting efforts should develop explicit approaches that incorporate additional expertise and analysis from scientists and public health officials. (The only documented systematic elicitation of expert judgment, for any type of modeling approach, in our review was the prediction market of Polgreen et al. [32].) Similar methods exist in weather and climate forecast (e.g., [50]). For example, meteorological forecasts are typically statistically post-processed to account for inherent model biases, and new methods for this post-processing are still being developed [51]. These combined results are then further vetted by meteorologists to monitor anomalous prediction behavior, and communicated to the public and decision makers. Infectious disease forecasting will need to explore and develop analogous frameworks for the post-processing of multiple forecast streams, the monitoring and calibration of these probabilistic forecasts, and the communication of these predictions to public health officials for decision support.

Fifth, now that diverse ILI forecasting approaches are available and some have demonstrated promising performance in validation studies, assessments of real-world applications could spur the transition of these approaches to public health practice. Pilot studies in health departments, medical facilities, or other settings could assess forecasting applications not only for predictive skill, but for user acceptance, contributions to public health decision-making, and other outcomes at the user-model interface. Evaluations should compare various modeling approaches on these key characteristics, to identify approaches useful (not just accurate) in real-world settings. For example, forecasting ILI time series or peak week could be useful for anticipating needed surge capacity of personnel and materials, but modeling methods that permit re-estimation of outcomes under various response scenarios could provide additional support to decision-makers.

Last, model developers and decision-makers must understand each other's work better. Developers are more likely to provide useful tools if they know the key decisions users will make in preparing for or responding to influenza outbreaks. They can develop and evaluate models around those specific decisions. To apply forecasting models effectively, decision-makers should become familiar with the modeling tools they might use, and understand their strengths, limitations, and key assumptions. Efforts to link modelers and public health officials through seminars, on-the-job observation, exercises, and other activities could foster this mutual understanding and improve collaboration during emergencies.
